# Primary Ovarian Mucinous Adenocarcinoma, Expansile Type, Misperceived As Pregnancy by the Patient

**DOI:** 10.7759/cureus.39077

**Published:** 2023-05-16

**Authors:** Christina Ortiz, Rachel Wexler, Katherine Drews-Elger, Ilya Fonarov, Damian Casadesus

**Affiliations:** 1 Hospital Medicine, Jackson Memorial Hospital, Miami, USA; 2 Pathology and Laboratory Medicine, Jackson Memorial Hospital, Miami, USA; 3 Internal Medicine, Jackson Memorial Hospital, Miami, USA

**Keywords:** histerectomy, ovarian tumour, pseudocyesis, ovarian adenocarcinoma, ovarian cancer

## Abstract

We present the case of a woman in her 20s with an eight-month history of increasing abdominal distention, dyspnea, and night sweats. The patient believed she was pregnant despite being told at another hospital that the pregnancy tests were negative, and no fetus was seen on an abdominal ultrasound. The patient delayed obtaining follow-up because of a distrust of the healthcare system and presented to our hospital at the behest of her mother. On physical examination, the abdomen was distended with a positive fluid wave, and a large mass was palpated in the abdomen. Gynecological examination was limited because of severe abdominal distension but a mass was palpable in the right adnexa. A pregnancy test and fetal ultrasound were performed, and the patient was not pregnant. A CT scan of the abdomen and pelvis revealed a large mass arising from the right adnexa. She underwent right salpingo-oophorectomy, appendectomy, omentectomy, lymph node dissection, and peritoneal implant resection. The biopsy confirmed intestinal-type IIB primary ovarian mucinous adenocarcinoma, expansile type, with peritoneal spread. Chemotherapy was provided for three cycles. A follow-up CT scan of the abdomen showed no evidence of a tumor six months after surgery.

## Introduction

Primary ovarian mucinous adenocarcinoma (OMAC) is a rare subtype of epithelial ovarian tumor that comprises 3% of all ovarian neoplasms [1,2]. Many of these tumors present as unilateral pelvic masses. The symptoms are more pronounced earlier in the disease, which allows for earlier detection and therefore increased survival rates. 

Primary OMAC has a favorable prognosis [1]. Mucinous tumors are generally diagnosed in patients who are younger than patients in whom other epithelial ovarian cancers are diagnosed. The prognosis depends on the stage of the disease. The five-year survival rate for mucinous adenocarcinoma localized to the ovary is greater than 90%. In contrast, metastatic mucinous adenocarcinoma, generally classified as either III or IV, has a median survival rate of between one and two years [2]. Low human chorionic gonadotropin (hCG), younger age, and lack of tobacco inhalation favor a better prognosis [3].

Currently, the best treatment method combines surgical procedures and adjuvant chemotherapy. Primary OMAC generally has a low sensitivity to chemotherapy, therefore treatment used consists of platinum agents and taxane [4]. 

Our patient had intestinal-type III primary OMAC with metastasis to the sigmoid colon, cecum, and peritoneum. More specifically, the histopathology of the tissue samples indicated an expansile type of mucinous adenocarcinoma. According to Morice et al., there have been only three cases of this type with peritoneal spread [2]. 

## Case presentation

A woman in her 20s with no past medical history presented to the emergency room at our hospital because of oligomenorrhea. The patient had visited another institution one month prior due to the loss of her menstrual period for seven months. She believed she was pregnant despite being told at another hospital that the pregnancy tests were negative, and confirming that no fetus was seen on an abdominal ultrasound. She delayed obtaining follow-up due to distrust of the healthcare system. The patient was depressed, her abdomen was distended, and she had informed her family members that she was pregnant. She visited our institution at her mother’s behest.

In our hospital, the patient complained of unintentional weight loss, right-sided abdominal pain, night sweats, chest pain, and shortness of breath. These symptoms were associated with abdominal distension and progressed over eight months. She endorsed irregular menses occurring every two months, lasting for about a week, and sometimes occurring twice in one month. Her last regular menstrual period was seven months before her medical evaluation. She denied any previous primary doctor visits, gynecological examinations, or oral contraceptive use. She was not sexually active, and she denied using tobacco or drugs. Her medications included ferrous sulfate, folic acid, and thiamine. Upon physical examination, the chest was tympanic to percussion and the lungs were clear to auscultation with decreased breath sounds bilaterally. The abdomen appeared distended with inversion of the umbilicus, was tense to palpation with a positive fluid wave, and had a palpable mass in the right lower abdomen. Gynecological examination revealed normal external genitalia; normal vaginal mucosae; and the cervix was closed, smooth, firm, midline, and without lesions or motion tenderness. The bimanual examination was limited because of abdominal distension but a mass was palpable in the right adnexa without tenderness. The patient refused a digital rectal exam. She underwent a urine human chorionic gonadotropin (hCG) qualitative pregnancy test, fetal US, and transvaginal US that confirmed she was not pregnant. The relevant laboratory study results are presented in Table 1.

**Table 1:** Relevant laboratory results at the time of admission AST: Aspartate aminotransferase, CEA: Carcinoembryonic antigen, CA: Cancer antigen, LDH: Lactate dehydrogenase, hCG: Human chorionic gonadotropin

Test	Patients' value	Normal value
Potassium	4.6 mml/L	3.6-5
Creatinine	0.50 mg/dL	0.52-1.04
Total bilirrubin	3.0 mg/ dL	0.2-1.3
AST	119 U/L	15-46
CA 19-9	1416 U/mL	0-37
CEA	49.9 ng/mL	0-2.5
CA 125	256 U/mL	0-35
hCG	<2.4 mlU/mL	<5
LDH	411 U/L	105-333

**Figure 1:**
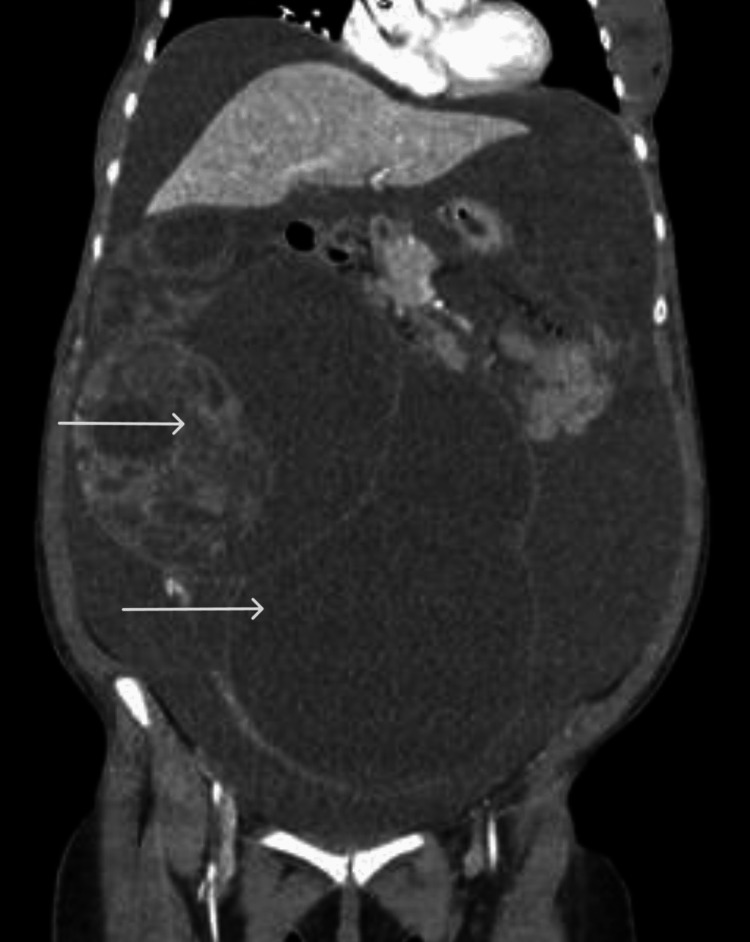
CT of the abdomen and pelvis revealed a cystic mass of the right adnexa

The patient underwent a gynecological US and CT scan of the abdomen and pelvis. The CT scan of the abdomen and pelvis revealed a large, complex cystic, irregular, multiloculated mass, with internal vascularity, measuring approximately 28 x 18 x 33 cm, with solid components arising from the right adnexa extending into the left adnexa and to the upper abdomen. Large-volume ascites were also noted (Figure 1). The gynecological US revealed a large complex cystic multiloculated mass with internal vascularity at the right adnexa and a large amount of free pelvic fluid. Our institution does not provide an ovarian-adnexal reporting and data system (O-RADS) score but the findings were consistent with O-RADS 5. Typically, correlation with multiphasic MRI with contrast is done for better characterization of ovarian tumors. However, because the tumor was so advanced, the MRI was not deemed necessary.

**Figure 2:**
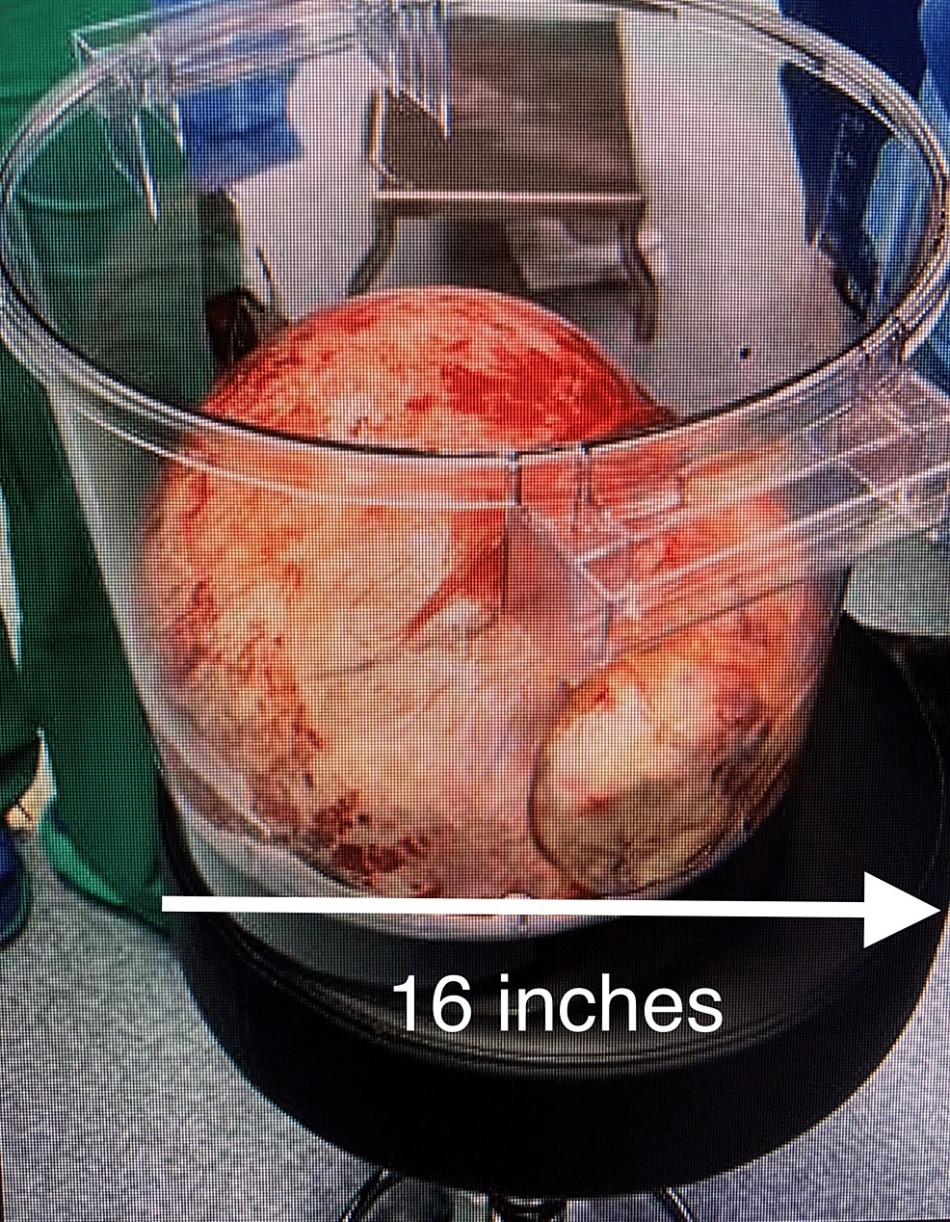
Ovarian mass removed during the right salpingo-oophorectomy

The CT scan of the abdomen and pelvis and the gynecological US suggested gynecological malignancy and an exploratory laparotomy was recommended. The patient underwent a paracentesis with the removal of 7.5 L of sanguineous fluid. Exploratory laparotomy showed a large multicystic right adnexal mass extending from the adnexa to the infra hepatic space, but the left adnexa and uterus were normal. Friable noninvasive peritoneal implants were present on the right pelvic sidewall, cecum, sigmoid colon, and cul-de-sac. The omentum was adherent to the abdominal wall. The patient underwent right salpingo-oophorectomy to preserve future fertility, as well as appendectomy, omentectomy, right pelvic and para-aortic lymph node dissection, and peritoneal implant resection (Figure 2).

**Figure 3:**
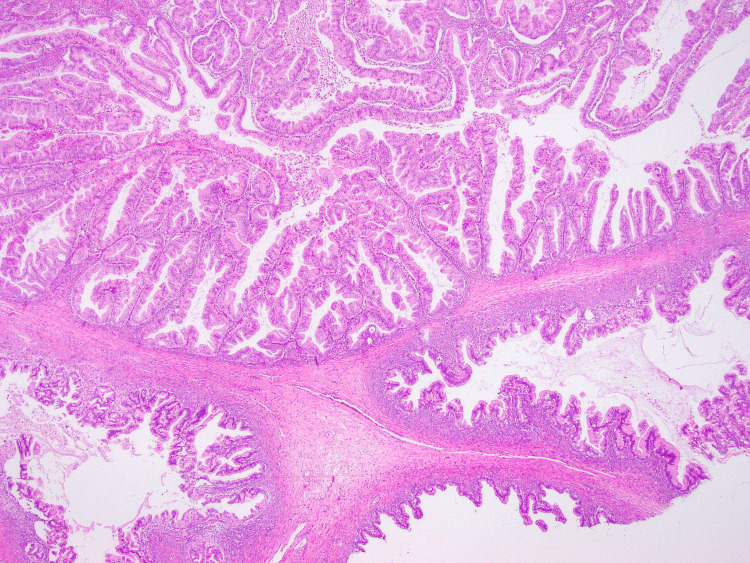
Mucinous adenocarcinoma intestinal type IIB (hematoxylin and eosin 2x)

**Figure 4:**
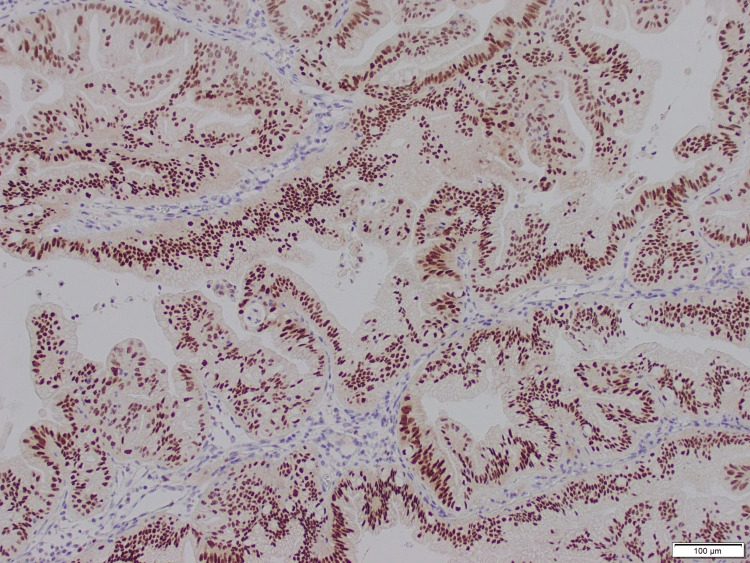
PAX8 immunostaining showed positive nuclear staining in tumor cells (2x) PAX8: Paired box gene 8

**Figure 5:**
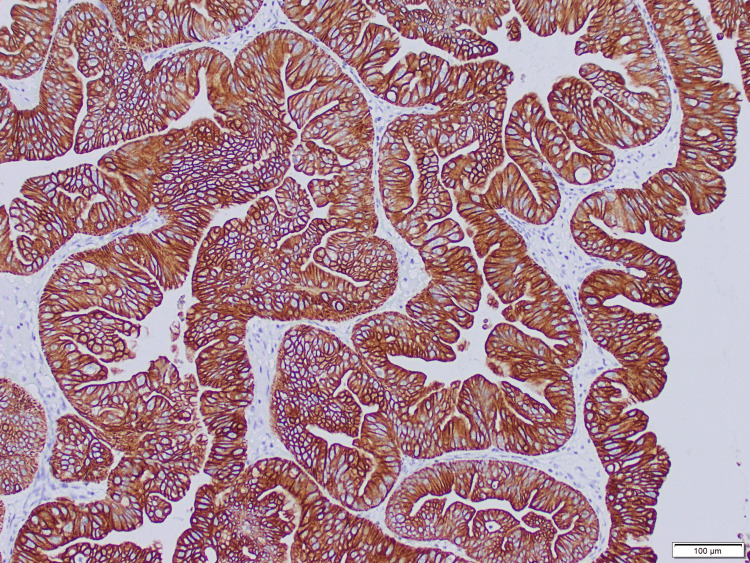
CK7 immunostaining showed diffuse cytoplasmic staining in tumor cells (2x) CK: Cytokeratin

The pathology results revealed an intestinal-type IIB primary OMAC (Figure 3). Paired box gene 8 (PAX8) and cytokeratin 7 (CK7) immunostaining showed positive nuclear staining in tumor cells (Figure 4), and diffused cytoplasmic staining in nuclear cells (Figure 5).

After removing the tumor, the patient’s appetite dramatically increased. Prior to chemotherapy the patient was counseled on the risks of infertility and recommended to undergo oocyte retrieval. Eleven oocytes were successfully retrieved.

In the outpatient clinic, the patient received three cycles of carboplatin and paclitaxel to decrease possible recurrence. Six months after surgery, the laboratory studies showed a normal cancer antigen (CA) 19-9, CA 125, and carcinoembryonic antigen (CEA). A follow-up CT scan of the abdomen and pelvis revealed no evidence of a tumor. After discharge from our facility, we followed up with the patient multiple times to see her progression through treatment and she was asymptomatic.

## Discussion

It is unknown why our patient developed such a rare tumor at a young age. Further genetic testing can provide better insight into the etiology of her cancer and other possible associated diseases. According to Babaier et al., Kirsten rat sarcoma virus (KRAS) mutations are implicated in half of all OMAC [3]. While genetic testing should not be limited to KRAS, identifying these mutations can allow healthcare professionals to create a more specific treatment plan.

Out of the approximately 230,000 new cases of ovarian cancer every year, primary OMAC accounts for only 3% of these cases [2]. In an analysis of data collected from the Surveillance, Epidemiology, and End Results Cancer Registry, approximately 26% of the diagnosed mucinous adenocarcinomas were found in women who were younger than 44 years old [2]. Women with these tumors are often diagnosed in the early stages, grades I and II, as the primary tumors are usually prominent and noticeable. About 1% of women are diagnosed at more advanced stages III and IV [5]. Guoy et al. in a retrospective analysis of stage I primary ovarian mucinous carcinoma described a better overall survival and disease progression survival in patients with the expansile subtype [6].

The incidence of primary OMAC has been decreasing since 2013 due to a better understanding of the differences between primary ovarian neoplasms and neoplasms resulting from metastatic cancer. One hypothesized reason for this decrease is better histological comprehension from pathologists to differentiate between the two etiologies [7]. It may be difficult to differentiate metastatic mucinous carcinomas from primary ovarian neoplasms. To differentiate primary and metastatic ovarian neoplasm, a combination of clinical, morphological, and immunohistochemical information should be considered. Compared to metastatic lesions, primary ovarian neoplasms tend to be unilateral, do not involve the surface, are negative in lymphovascular invasion, lack multinodularity, have an area larger in size (>13cms), and in general exhibit an expansile rather than invasive growth pattern. Primary ovarian neoplasms may acquire a gastrointestinal phenotype and loose gynecologic-specific markers such as PAX8 and estrogen receptors. Therefore the use of additional immunohistochemistry markers becomes necessary to identify the site of origin [7]. Most primary ovarian mucinous tumors express PAX8 and CK7 immunostaining [8, 9]. They can also demonstrate focal CK20 and CDX-2 labeling. 

Even though the incidence of primary OMAC has decreased, it is still essential to have regular gynecological examinations. The US Preventative Task Force currently recommends against screening in asymptomatic women who do not have a high risk of developing ovarian cancer [10]. The American College of Obstetricians and Gynaecologists recommends that female visits to gynecologists start between ages 13 and 15 [11]. Regular gynecological exams ensure that any ominous symptoms or growths can be treated in an appropriate and timely manner. In this case, the patient delayed receiving the appropriate gynecological care due to a false belief and distrust of the healthcare system. Proper counseling is needed to ensure that patients understand diagnostic test results, the treatment plan, and the importance of follow-up care.

## Conclusions

Patients who suspect pregnancy need adequate counseling and medical care at the time of the initial healthcare encounter. Our patient believed that she was pregnant despite being told that the pregnancy tests were negative, and no fetus was seen on an abdominal ultrasound. Being pregnant is life-changing for most women, and the emotional aspects of pregnancy may have contributed to the patient maintaining a false belief. In this case, the patient delayed accessing gynecological care promptly which could have placed the patient at risk of a poor outcome. This case highlights the importance of proper counseling and patient care to ensure that patients receive the appropriate follow-up. In addition, young women need to know the importance of regular gynaecological exams to ensure that any ominous symptoms or growths can be treated in an appropriate and timely manner.
